# Research progress of *CMTM4* in the tumor immune microenvironment and immunotherapy

**DOI:** 10.3389/fimmu.2026.1741477

**Published:** 2026-05-22

**Authors:** Xinyao Zhang, Qijiao Wang, Jie Li, Jing Cao, Zhijuan Guo, Ru Ji

**Affiliations:** 1 Inner Mongolia Medical University, Hohhot, China; 2 Tongliao People’s Hospital, Tongliao, China; 3 Inner Mongolia Medical University Affiliated Cancer Hospital, Hohhot, China

**Keywords:** biomarkers, CMTM4, immunotherapy, PD-1/PD-L1, tumor immune microenvironment

## Abstract

Immunosuppression within the tumor microenvironment (TME) remains a major barrier to durable responses to cancer immunotherapy. CKLF-like MARVEL transmembrane domain-containing 4 (*CMTM4*), a member of the CMTM family, has emerged as a regulator of tumor immune regulation and membrane protein trafficking. This critical narrative review summarizes the genetic and structural features of *CMTM4*, its immunological functions under physiological and pathological conditions, and its multifaceted roles within the immune compartment of the TME. The review focuses on three aspects (1): mechanisms by which *CMTM4* contributes to programmed death-ligand 1 (PD-L1) stability and interacts with TME-associated membrane proteins (2); cell-type-specific interactions between *CMTM4* and immune effector or suppressor cells; and (3) its potential value as a candidate prognostic biomarker and therapeutic target for modulating resistance to immune checkpoint inhibitors (ICIs). This review also discusses unresolved mechanistic questions, limitations of current research models, and challenges in clinical translation. Future studies integrating single-cell multi-omics, spatial transcriptomics, proteomics, and organoid models may help clarify the context-dependent functions of *CMTM4* and provide a stronger basis for its translational evaluation.

## 1 Introduction

According to the latest 2022 estimates from the International Agency for Research on Cancer (IARC), there were nearly 20 million new cancer cases worldwide (including non-melanoma skin cancer), and cancer-related deaths reached 9.7 million (1). The most common cancers include lung cancer (LC), breast cancer (BC) in women (11.6%), colorectal cancer (CRC) (9.6%), prostate cancer (7.3%), and gastric cancer (GC) (4.9%), highlighting the urgent need to develop effective treatment strategies and prognostic biomarkers. Traditional treatments (chemotherapy, radiation, surgery) and immunotherapies (e.g., ICIs) have achieved significant progress. However, over 60% of patients fail to achieve a durable response due to tumor heterogeneity, drug resistance, and an immunosuppressive TME (2, 3). The TME plays a key regulatory role in tumor initiation, progression, metastasis, and drug resistance, but the mechanisms by which its specific components influence tumor biological behavior remain incompletely understood.

Based on cellular composition, the TME can be divided into an immune microenvironment, dominated by immune cells, and a non-immune microenvironment, centered on fibroblasts. The immune compartment of the TME primarily comprises two major categories of immune cell populations: one consists of immune effector cells, such as CD4^+^ T cells, CD8^+^ cytotoxic T cells, and NK cells; the other consists of immunosuppressive cells, including regulatory T cells (Tregs), regulatory B cells (Bregs), M2 macrophages, and myeloid-derived suppressor cells (MDSCs) (4). The dynamic balance within the immune compartment of the TME directly influences patient prognosis. Tumor cells mediate immune evasion through complex interactions with the immune system, thereby driving tumor proliferation, invasion, metastasis, and the development of resistance to immunotherapy (5). Therefore, clarifying the molecular mechanisms that shape the immune compartment of the TME may support the development of more precise therapeutic strategies.

Recent studies have revealed a close association between *CMTM4* and the TME as well as its immune cells (6–11). The CMTM4 protein contains at least one conserved MARVEL domain, a structure composed of four transmembrane helices and cytoplasmic N- and C-terminal regions, which mediates biological processes such as vesicular transport, membrane fusion, and protein trafficking (12, 13). The CMTM family is widely expressed in the immune system; its functions were initially elucidated in autoimmune diseases, and subsequent research has gradually expanded into the field of oncology, where *CMTM3*, *CMTM5*, *CMTM7*, and *CMTM8* have been extensively studied in various malignant tumors (14–16). In contrast, research on *CMTM4* has lagged, yet its biological functions are highly diverse: it regulates the biosynthesis and transport of CXCR4 (17), modulates IL-17 receptor signaling in autoimmune diseases (18, 19), maintains intestinal homeostasis (20), participates in male reproduction (21), and contributes to physiological processes such as angiogenesis by regulating the recycling of vascular endothelial cadherin (22).

In 2017, two landmark studies published in *Nature* independently confirmed that *CMTM4* is a regulator of PD-L1 protein stability (23, 24). This discovery directly links *CMTM4* to the PD-1/PD-L1 immune checkpoint pathway, one of the most clinically successful targets in cancer immunotherapy, and suggests that *CMTM4* may represent a potential molecular target for modulating ICI resistance (25, 26). Subsequent studies have reported that *CMTM4* contributes to PD-L1 protein stability or membrane expression in selected cancer models. Additionally, it contributes to the remodeling of the TME by interacting with multiple immune cells, regulating relevant signaling pathways, and influencing cancer stem cell properties. Its expression levels are also tightly regulated by non-coding RNAs and epigenetic modifications (27–30), further highlighting the complexity of its functions in tumor biology.

This review provides a critical narrative synthesis of representative studies on *CMTM4* in tumor immune regulation and immunotherapy. It focuses on the roles of *CMTM4* in TME remodeling, immune-cell interactions, PD-L1 stabilization, and tumor immune evasion. This article was not conducted as a PRISMA-based systematic review or meta-analysis.

## 2 *CMTM4*: from fundamental immune modulator to an important molecule in tumor immunity

### 2.1 Genetic and protein structural features

*CMTM4* is a member of the CMTM family, which is named for its fusion of chemokine-like factor (*CKLF*) sequences with MARVEL transmembrane domains and consists of *CKLF* and *CMTM1–CMTM8* (12). The *CMTM4* gene is located on chromosome 16q22.1 and forms a gene cluster with *CKLF* and *CMTM1–3*; this region contains multiple tumor suppressor genes (31).

Among the CMTM family, the *CMTM4* sequence is the most conserved. Similar to its family members, the *CMTM4* precursor mRNA has three splice variants: *CMTM4-V1* encodes 234 amino acids, *CMTM4-V2* encodes 208 amino acids, and *CMTM4-V3* encodes 179 amino acids. Among these, *CMTM4-V1* and *CMTM4-V2* are widely expressed in various human tissues and cancer cell lines, whereas *CMTM4-V3* is specifically expressed only in kidney and placental tissues (32–34). CMTM4 protein is most highly expressed in the thyroid, followed by the brain, kidneys, and colon, while its expression is lower in the liver, spleen, lymph nodes, and bone marrow. The CMTM4 protein possesses structural features of both classic chemokines and the transmembrane 4 superfamily (TM4SF), and can influence tumor cell proliferation and invasion by activating and chemotaxing immune cells (2). The MARVEL domain of the CMTM4 protein mediates its localization to the plasma membrane, Golgi membrane, and intracellular vesicles, and participates in regulating transmembrane transport and the formation of tight junctions. This provides the structural basis for its subsequent role in regulating the cycling and stability of key membrane proteins such as PD-L1 (12).

### 2.2 Core immune functions

*CMTM4* plays a regulatory role in the immune system and is highly expressed primarily in immune-related tissues and cells, such as the bone marrow and peripheral blood cells. Its expression is particularly prominent in quiescent CD19^+^ cells and activated peripheral blood mononuclear cells, providing the foundation for its immune regulatory functions (3).

As a multifunctional immune regulatory molecule, *CMTM4* is deeply involved in both innate and adaptive immunity by regulating key immune signals, maintaining immune homeostasis, and participating in inflammatory responses; its functional abnormalities are closely associated with various immune-related diseases. Studies have shown that *CMTM4* is a regulator of CXCR4 biosynthesis and trafficking. By modulating its glycosylation, transport from the endoplasmic reticulum to the cell membrane, and ligand-dependent degradation, it influences CXCR4-mediated cell migration and the activation of the AKT signaling pathway (17).

More importantly, studies have found that *CMTM4* is a core component of the interleukin-17 receptor (IL-17R) signaling complex. It directly interacts with the IL-17RC subunit, maintaining its stability and membrane localization, thereby precisely regulating IL-17-mediated NF-κB pathway activation and neutrophil migration, directly influencing the balance of immune responses at sites of inflammation or infection (18). This function has been validated in multiple contexts: regulating neutrophil migration in lymphoendothelial cells (19); maintaining intestinal immune homeostasis via the IL-17 signaling axis and the gut microbiota, with its absence exacerbating colitis (20).

Furthermore, *CMTM4* participates in the immune regulation of sepsis; it can indirectly regulate PD-L1 expression through STAT2 phosphorylation, influencing macrophage apoptosis (35); its promoter methylation exhibits differences in autoimmune diseases such as systemic lupus erythematosus (SLE) and rheumatoid arthritis (RA), and is associated with SLE genetic variants, suggesting its involvement in the pathogenesis of autoimmune diseases (36, 37).

### 2.3 The bridge to tumor immunology

**Figure 1:**
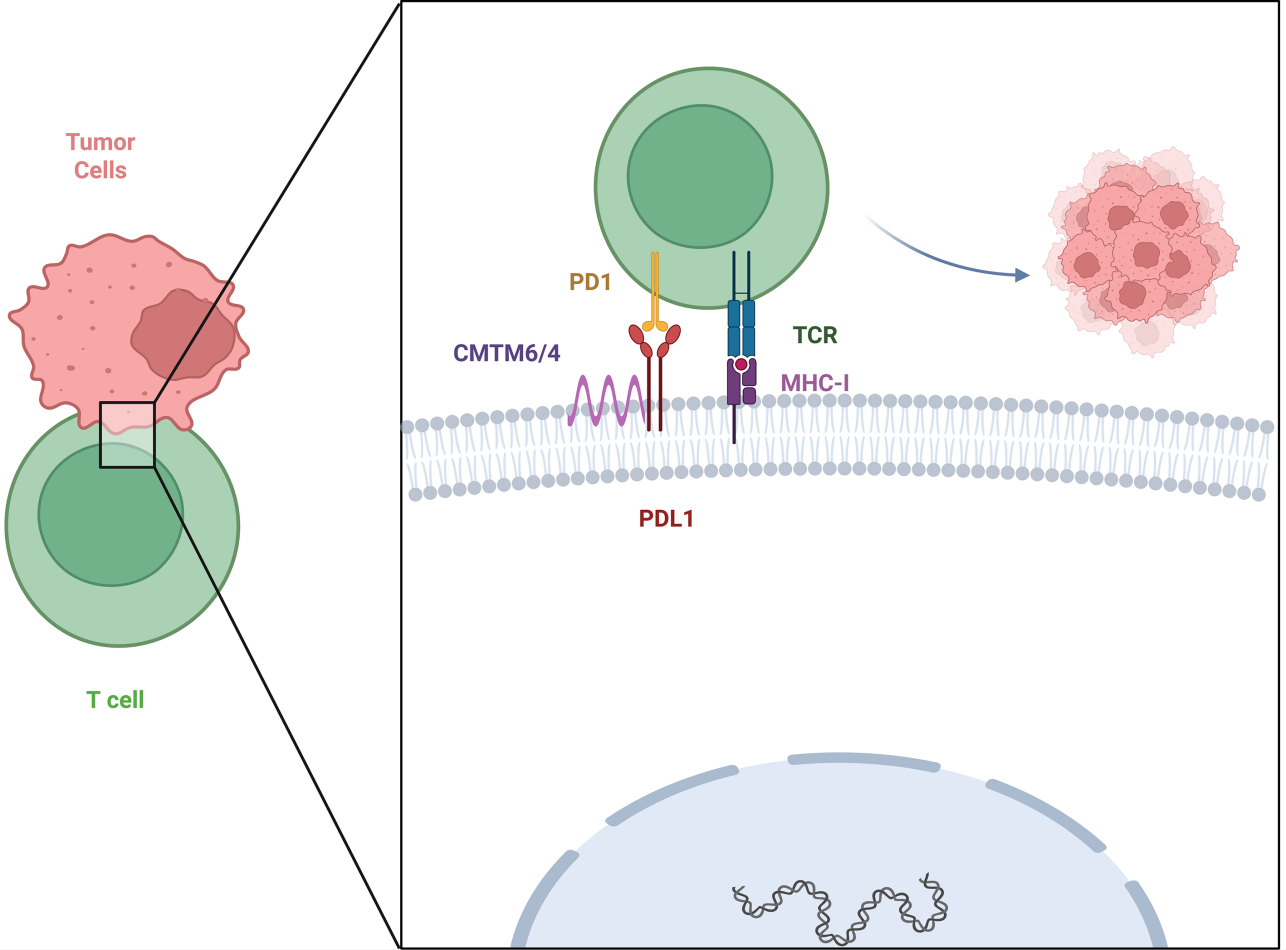
CMTM4/CMTM6-mediated regulation of PD-L1 stability. CMTM6 is generally considered a dominant regulator of PD-L1 membrane stability in many tumor models, whereas CMTM4 may act as a compensatory regulator in CMTM6-deficient or CMTM6-low contexts. By contributing to PD-L1 stabilization and reducing its degradation, CMTM4 may help maintain PD-L1 surface expression and promote PD-1/PD-L1-mediated T-cell dysfunction. This mechanism should be interpreted in a tumor-type- and context-dependent manner.

The focus of *CMTM4* research has gradually shifted from the field of basic immunology—where it regulates key immune signals in inflammation, autoimmune diseases, and various physiological processes—to the field of tumor immunology. This pivotal shift stems from its identification as a regulator of PD-L1, which is a central mediator of tumor immune evasion. Breakthrough research published in *Nature* has confirmed that *CMTM4* is a key upstream regulator of PD-L1 (23, 24, 38). As summarized in **Figure 1**, *CMTM4* should not be interpreted as an isolated PD-L1 regulator. Instead, its function should be considered within the broader *CMTM4/CMTM6* regulatory axis. *CMTM6* appears to act as the dominant PD-L1 stabilizer in many tumor models, whereas *CMTM4* may function as a compensatory regulator in *CMTM6*-deficient or *CMTM6*-low contexts. Therefore, **Figure 1** emphasizes not only PD-L1 stabilization but also the functional redundancy and potential compensation between *CMTM4* and *CMTM6.* This links *CMTM4* to the clinically successful PD-1/PD-L1 pathway, establishing *CMTM4* as a molecular bridge connecting basic immune function with tumor immune evasion.

The fundamental role of *CMTM4* in inflammation and immune homeostasis provides a crucial basis for its involvement in tumorigenesis and tumor progression. Studies have shown that *CMTM4* maintains intestinal immune homeostasis through the IL-17 signaling-S100A8/A9-RAGE axis and gut microbiota regulation; its deficiency can lead to dysbiosis and upregulation of pro-inflammatory factors, exacerbating colitis, suggesting that *CMTM4* plays a role in chronic inflammation-related diseases (20). In sepsis models, *CMTM4* within macrophages indirectly regulates PD-L1 expression via a STAT2-dependent pathway and participates in apoptosis; this mechanism provides crucial evidence for understanding its immunoregulatory function in tumor-associated macrophages (TAMs) (35).

More importantly, the fundamental immunological functions of *CMTM4* can directly influence tumor initiation. In GC, *CMTM4* deficiency reduces DNA damage and inhibits pseudopyloric metaplasia and gastric carcinogenesis by downregulating the IL-17 signaling pathway, decreasing IL-17RC expression, and suppressing NF-κB activity and NOX1 levels (39). This finding further demonstrates that *CMTM4* not only participates in inflammation and immune regulation but can also directly influence tumor initiation and malignant transformation processes.

In summary, *CMTM4* links basic immune regulation with tumor immune evasion through its roles in membrane protein trafficking, inflammatory signaling, PD-L1 stabilization, and remodeling of the immune compartment of the TME. These findings provide the rationale for further discussing its functions in the TME and its potential translational relevance.

## 3 Research progress on CMTM4 and PD-1/PD-L1

The PD-1/PD-L1 immune checkpoint pathway is central to tumor immune evasion, as its engagement suppresses T-cell activation and facilitates tumor progression (25, 26, 40–42). Although targeting this pathway with ICIs has transformed cancer therapy, resistance remains a major clinical hurdle (43, 44). The recent discovery of *CMTM4* and *CMTM6* as regulators of PD-L1 stability has provided critical insights into overcoming ICI resistance and underscored the importance of understanding PD-L1 regulatory networks.

Burr et al. (23) and Mezzadra et al. (24) reported the regulation of PD-L1 by *CMTM6* and *CMTM4*, respectively, in *Nature*. Burr et al. found that *CMTM6* stabilizes the membrane expression of PD-L1 by binding to PD-L1 and blocking its lysosomal degradation. This mechanism does not rely on the secretory transport of PD-L1 but rather prevents its degradation via the lysosomal pathway by promoting the endocytic recycling of PD-L1. Genome-wide screening revealed that *CMTM6* exhibits high selectivity for PD-L1; knocking out *CMTM6* leads to downregulation of PD-L1 but does not affect the expression of MHC I or PD-L2 molecules, thereby significantly enhancing T-cell activation and anti-tumor effects. Mezzadra et al. identified CMTM4, the closest homolog of CMTM6 sharing 55% amino acid homology, as a regulator of PD-L1 expression in a CMTM6-deficiency-dependent manner. This functional feature has been verified in a panel of tumor cell lines covering multiple cancer types, including melanoma (A375), non-small cell lung cancer (NSCLC, H2030 cells), thyroid cancer (8505C) and CRC (RKO). In most of these cellular models, CMTM4 mainly exerts a compensatory regulatory effect on PD-L1 protein stability upon CMTM6 depletion or knockdown, while exhibiting negligible regulatory activity in cells with intact endogenous CMTM6 expression. Importantly, this compensatory regulatory mode is not universal. In certain tumor models with high basal CMTM4 expression (e.g., H2030 cells), CMTM4 also exhibits a partial CMTM6-independent basal regulatory activity, modulating PD-L1 levels even when CMTM6 is normally expressed (24). *CMTM6* and *CMTM4* can stabilize PD-L1 by binding to it, reducing STUB1-mediated ubiquitination and prolonging its half-life (38).

CMTM4 versus CMTM6: Redundancy, Compensation, and Therapeutic Implications.

Although *CMTM4* and *CMTM6* both contribute to PD-L1 protein stability, their functional hierarchy and therapeutic implications are not identical. As described above, *CMTM6* appears to act as the dominant PD-L1 stabilizer in many tumor models, whereas *CMTM4* often functions as a compensatory regulator in *CMTM6*-deficient or CMTM6-low contexts. This redundancy suggests that *CMTM4*-directed intervention alone may have limited effects in tumors where CMTM6 remains the major PD-L1-stabilizing molecule. Conversely, *CMTM4* may become more functionally relevant in tumors with reduced *CMTM6* activity or in tumors where CMTM4 regulates immune suppression through PD-L1-independent mechanisms, such as IL-17 receptor signaling, epidermal growth factor receptor trafficking, myeloid-derived suppressor cell recruitment, macrophage polarization, or radiotherapy resistance. Therefore, *CMTM4* and *CMTM6* should not be treated as interchangeable targets. Future studies should evaluate the *CMTM4/CMTM6* expression ratio, PD-L1 dependency, epithelial versus stromal localization, and immune infiltration status when considering CMTM4-related therapeutic strategies.

**Figure 2:**
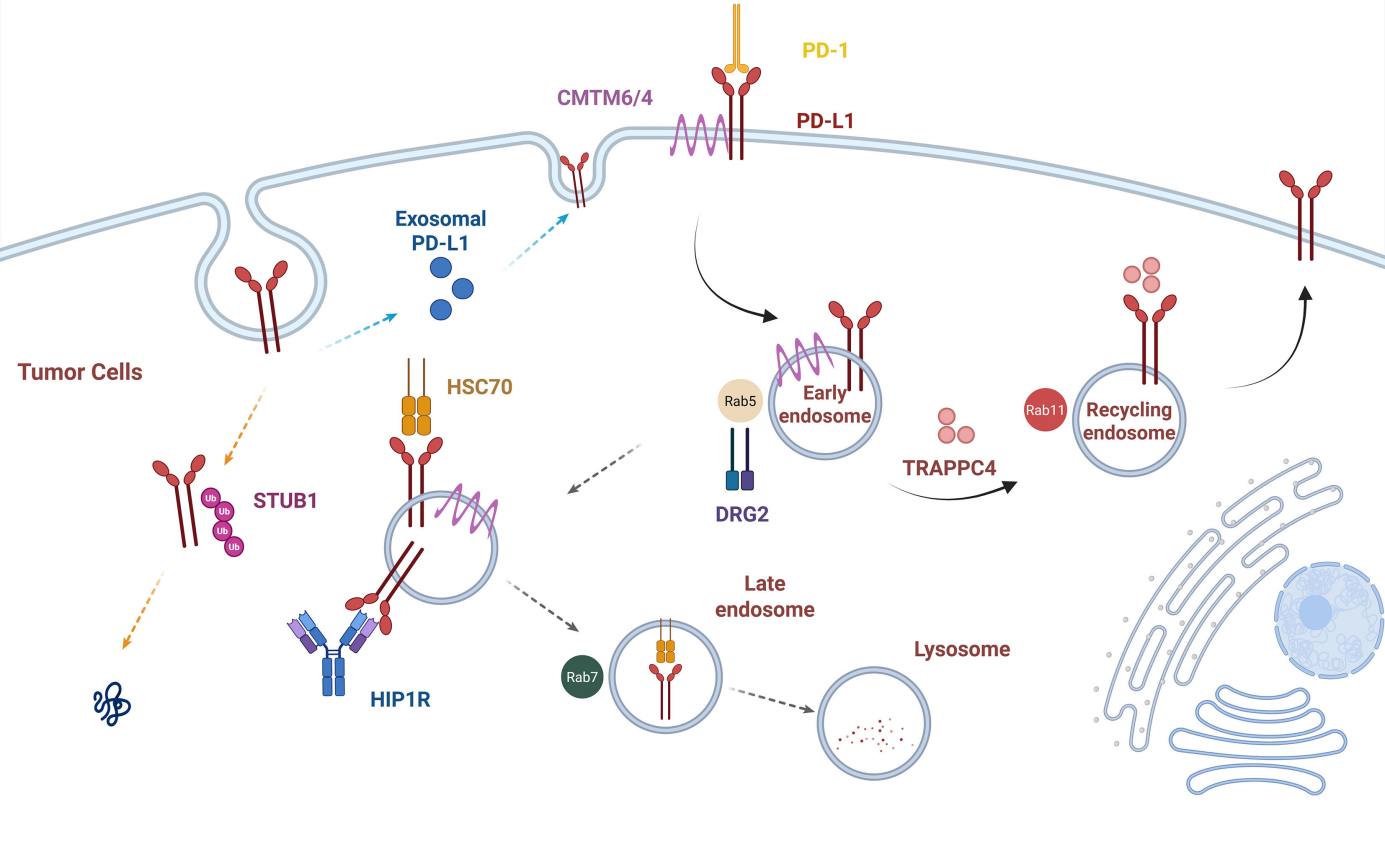
Intracellular trafficking and degradation network of PD-L1. PD-L1 surface abundance is controlled by endocytic recycling, lysosomal degradation, proteasomal degradation, and exosomal release. CMTM4 and CMTM6 participate in this broader trafficking network by contributing to PD-L1 stability and membrane localization. Other trafficking-related molecules, including TRAPPC4, HIP1R, DRG2, and Hsc70, may also influence PD-L1 localization and degradation. This figure highlights that PD-L1 regulation is a dynamic membrane trafficking process rather than a single-step pathway.

Upon internalization, if PD-L1 is not recycled to the plasma membrane, its primary fate is degradation via the lysosomal pathway or secretion in the form of exosomes (45). Research has identified trafficking protein particle complex subunit 4 (TRAPPC4) as an interacting protein of PD-L1. Its function is to act as a key linker molecule, mediating the interaction between PD-L1 and the Rab11 GTPase on circulating endosomes, thereby driving the recycling process of internalized PD-L1, allowing it to return to the plasma membrane and evade lysosomal degradation. However, whether this process is regulated by CMTM4/6 remains to be elucidated (46, 47). In contrast, Huntingtin-interacting protein 1-related protein (HIP1R) binds to PD-L1 via its intracellular di-leucine endosomal motif and, as a key haptomer, directly mediates the lysosomal degradation pathway of PD-L1 (48). Another study revealed that DRG2 influences PD-L1 endosomal trafficking by regulating the activity of Rab5 GTPase: in the presence of DRG2, Rab5 is normally inactivated, promoting the transport of PD-L1 from Rab5-positive early endosomes to Rab11-positive recycling endosomes, thereby maintaining its cell membrane localization (49). Furthermore, the chaperone protein Hsc70 has been reported to participate in the lysosomal degradation pathway of PD-L1 and competitively binds to PD-L1 with CMTM6; interestingly, overexpression of Hsc70 in *CMTM6*-deficient cells effectively prevents the degradation and loss of PD-L1 (50). **Figure 2** places CMTM4-mediated PD-L1 stabilization within the broader intracellular trafficking network of PD-L1. PD-L1 surface abundance is determined by the balance among endocytic recycling, lysosomal degradation, proteasomal degradation, and exosomal release. Therefore, *CMTM4* should not be viewed as a single linear regulator of PD-L1, but rather as one component of a membrane-trafficking system that controls the duration and intensity of PD-1/PD-L1-mediated immune suppression. In summary, available experimental evidence suggests that *CMTM4* may enhance the ability of PD-L1-expressing tumor cells to suppress T cells.

Multiple studies across different cancer types have suggested that *CMTM4* may contribute to PD-L1 regulation and may have clinical relevance in selected contexts. In type I renal cell carcinoma (RCC), *CMTM4* expression levels are significantly higher than in types II and III, and patients with type I RCC show poorer responses to ICIs such as CTLA-4 and PD-1/PD-L1 inhibitors, suggesting that *CMTM4* may serve as a potential therapeutic target for patients who are unresponsive to ICIs (51). In GC, the proportion of *CMTM4*-positive cells in the tumor epithelium is significantly higher than that in the adjacent peritumoral tissue. The co-expression relationship between *CMTM4* and PD-L1 exhibits significant spatial specificity. In the tumor epithelium, the expression of these two proteins is positively correlated, and they colocalize on the tumor cell membrane; together with *CMTM6*, they constitute a predictive biomarker for a better response to anti-PD-1/PD-L1 immunotherapy. However, in the stromal compartment, *CMTM4* expression is negatively correlated with PD-L1 expression (52). This finding does not necessarily indicate that *CMTM4* directly antagonizes PD-L1-mediated immune suppression in the stroma. Rather, it suggests that the *CMTM4*–PD-L1 relationship may be spatially and cell-type dependent. These apparently contradictory findings should be interpreted cautiously. First, most available clinical observations are based on correlative tissue analyses rather than cell-type-specific functional experiments; therefore, they cannot directly prove that *CMTM4* regulates PD-L1 in stromal cells. Second, stromal PD-L1 expression may be driven predominantly by inflammatory cytokines, such as IFN-γ, or by the abundance of specific immune-cell subsets, rather than by *CMTM4*-dependent post-translational stabilization. Third, *CMTM6*, which is generally considered a dominant stabilizer of PD-L1 in many tumor models, may confound the interpretation of *CMTM4*-specific effects if it is not evaluated simultaneously. Fourth, *CMTM4* may exert PD-L1-independent functions in stromal cells, including regulation of membrane trafficking, IL-17 receptor signaling, immune-cell recruitment, or extracellular matrix-associated remodeling. Therefore, *CMTM4* should not be regarded as a universal PD-L1 stabilizer across all tumor compartments. Its biological significance should be interpreted according to tumor type, cellular source, spatial localization, *CMTM6* status, and inflammatory context. Future studies using spatial transcriptomics, multiplex immunofluorescence, single-cell sequencing, and cell-type-specific functional experiments are needed to clarify whether *CMTM4* acts as a checkpoint regulator, a stromal immune-context marker, or an independent regulator of tumor immunity.

In hepatocellular carcinoma (HCC) and intrahepatic cholangiocarcinoma (ICC), the regulatory role of *CMTM4* on PD-L1 has been further confirmed. *CMTM4* deficiency significantly reduces cell surface PD-L1 expression, regardless of IFN-γ stimulation, supporting the role of *CMTM4* as a positive regulator of PD-L1 in these experimental models (7). This finding is consistent with the report by Mezzadra et al., who demonstrated that *CMTM4* suppresses PD-L1 expression in an IFN-γ-independent manner; specifically, *CMTM4* prevents PD-L1 degradation via both the endolysosomal and proteasomal pathways. This study did not explicitly address the impact of CMTM6 expression levels on the regulatory role of CMTM4; whether its conclusions depend on the absence of CMTM6 requires further verification.

In other cancer types, the regulatory network of *CMTM4* is also being gradually elucidated. In CRC, the circular RNA CDR1-AS promotes the gene expression of *CMTM4* and *CMTM6* through a mechanism independent of miR-7, thereby enhancing the levels of PD-L1 protein on the cell surface (53, 54). Furthermore, metabolic interventions have been found to influence the *CMTM4*-PD-L1 axis, for instance, it has been reported that a ketogenic diet, by altering the body’s energy metabolism, activates the AMPK protein kinase. This kinase blocks the binding of PD-L1 to the stabilizing molecule CMTM4 through phosphorylation at the Ser283 site of the PD-L1 protein, thereby promoting PD-L1 degradation and enhancing the efficacy of anti-CTLA-4 immunotherapy (55).

The following sections therefore focus not on repeating the molecular details of PD-L1 stabilization, but on how *CMTM4*-related pathways shape immune-cell behavior and influence therapeutic responses in different tumor contexts.

## 4 Recent advances in the interaction between CMTM4 and immune cells in the TME

### 4.1 Macrophage

TAMs are among the most abundant immune cells in the TME, and their dynamic plasticity plays a decisive role in tumor progression and treatment response (56, 57). Single-cell studies have further revealed the remarkable functional diversity of TAMs across different cancer types; these cells drive malignant progression by promoting immune suppression, angiogenesis, and tissue remodeling (58).

**Figure 3:**
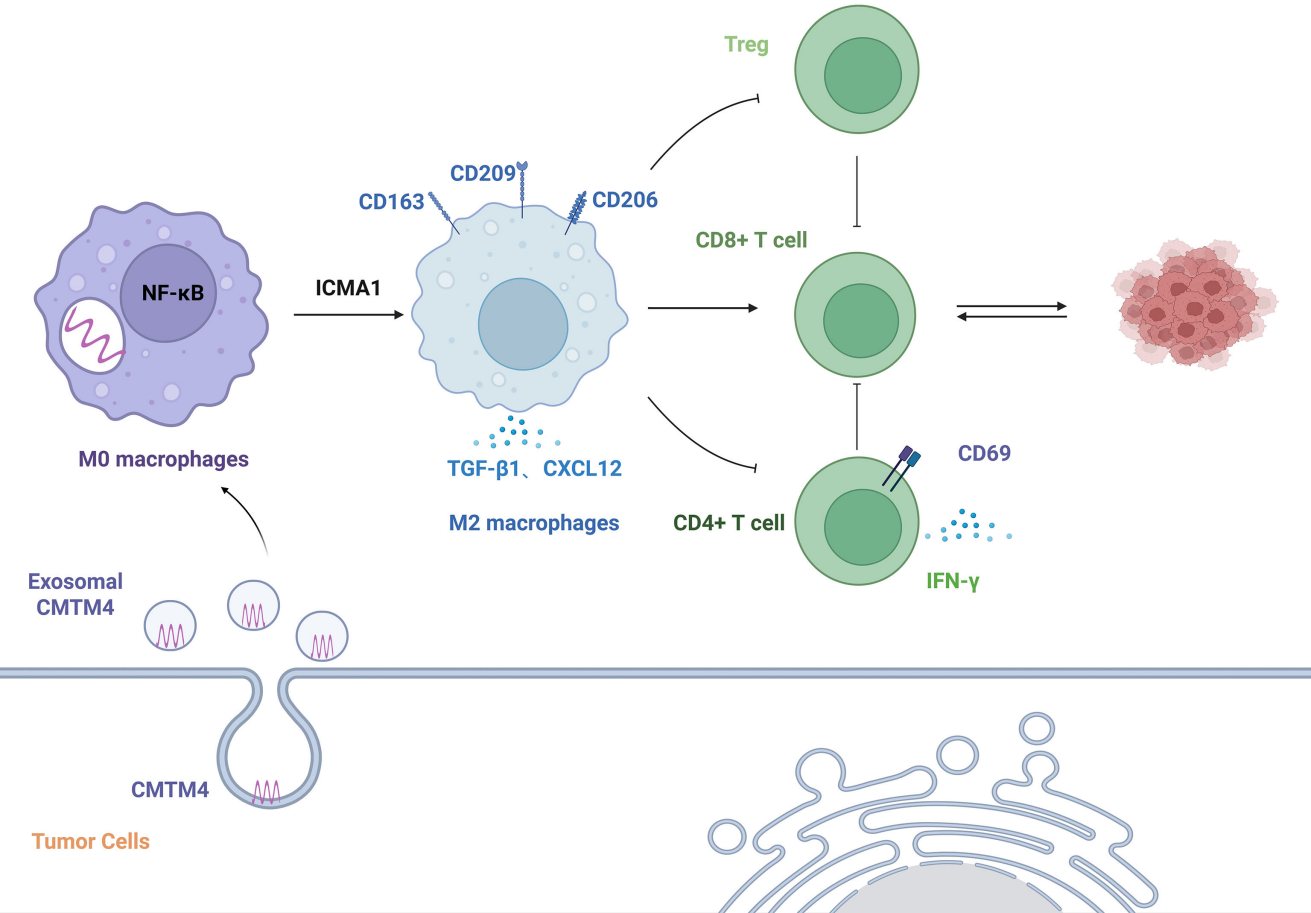
CMTM4-mediated regulation of tumor-associated macrophages. Tumor-derived CMTM4, including exosomal CMTM4 in ovarian cancer models, may promote immunosuppressive TAM phenotypes and enhance secretion of immunosuppressive mediators such as TGF-β1 and CXCL12. This macrophage-centered mechanism contributes to T-cell suppression and tumor immune escape, indicating that CMTM4 may regulate the TME through PD-L1-independent myeloid-cell remodeling.

In recent years, *CMTM4* has been identified as an emerging key factor regulating the phenotype and function of TAMs. In the brain glioma microenvironment, the co-expression of *CMTM4* and PD-L1 in TAMs induces immune suppression, significantly impairs the anti-tumor function of T cells, and is associated with poor patient prognosis (27). Multiplex immunofluorescence staining confirmed the colocalization of both molecules in CD68^+^ macrophages; however, no detailed macrophage subtyping was performed. Future studies could validate whether *CMTM4* is specifically overexpressed in M2 macrophages by co-labeling with typical markers such as CD68 and CD163 (2). In ovarian cancer (OC), the CMTM4 protein in tumor-derived exosomes enhances the secretion of immunosuppressive cytokines such as TGF-β1 and CXCL12 by activating the NF-κB pathway in TAMs. Concurrently, *CMTM4*-mediated upregulation of ICAM1 enhances adhesion and communication between cancer cells and macrophages, synergistically promoting macrophage polarization toward the M2 phenotype, suppressing CD8^+^ and CD4^+^ T cell function, upregulating Tregs, and reducing CD69 expression and IFN-γ secretion, ultimately driving tumor immune evasion and metastasis (9). As illustrated in **Figure 3**, *CMTM4*-mediated macrophage modulation represents a PD-L1-independent mechanism of tumor immune escape. In ovarian cancer models, tumor-derived exosomal *CMTM4* promotes immunosuppressive TAM phenotypes, increases the secretion of immunosuppressive mediators, and contributes to impaired T-cell activity. This mechanism expands the biological significance of *CMTM4* beyond PD-L1 stabilization and suggests that *CMTM4* may remodel the TME through myeloid-cell reprogramming. A comprehensive analysis of the CMTM family and immune infiltration in esophageal carcinoma (ESCA) revealed that *CMTM4* expression is significantly positively correlated with the M2 macrophage markers CD163 and MS4A4A, suggesting that *CMTM4* participates in tumor progression by regulating M2 macrophage infiltration in the ESCA immune microenvironment (11). In sepsis, *CMTM4* expression is elevated in macrophages, and *CMTM4* inhibition reduces macrophage apoptosis. Mechanistically, this effect appears to involve STAT2-dependent checkpoint signaling rather than direct binding between *CMTM4* and PD-L1. Although this evidence is derived from a non-tumor inflammatory context, it may provide a useful reference for understanding *CMTM4*-related macrophage regulation in the TME (35).

### 4.2 CD8^+^ T cells

CD8^+^ T cells are the ultimate effector cells of anti-tumor immunity, and their infiltration depth and functional status are among the most important biomarkers for predicting the efficacy of ICIs (59, 60). However, the TME can induce CD8^+^ T cells to enter a state of functional exhaustion, characterized by persistent expression of inhibitory receptors (such as PD-1) and progressive loss of effector function (61).

As discussed above, *CMTM4* may indirectly contribute to CD8^+^ T-cell dysfunction in selected tumor contexts (7). Cross-cancer analyses show that *CMTM4* is highly expressed in tumors with poor CD8^+^ T-cell infiltration and is negatively correlated with T-cell infiltration markers (8, 62). Similar negative associations have also been reported in locally advanced rectal cancer (LARC) and pleural mesothelioma (PM) (63, 64). In HCC models, *CMTM4* knockdown increases CD8^+^ T-cell infiltration and enhances anti-tumor immunity (7). Most studies show that the level of CD8^+^ T-cell infiltration in HCC is significantly negatively correlated with *CMTM4* mRNA expression, suggesting that *CMTM4* may inhibit the activity or recruitment of immune cells (6, 65, 66). In LC and BC, *CMTM4* promotes the secretion of inflammatory factors such as G-CSF by tumor cells through the internalization and stabilization of EGFR on the cell membrane, thereby recruiting and polarizing PMN-MDSCs. This significantly impairs the tumor-infiltrating capacity and cytotoxic function of CD8^+^ T cells, thereby establishing an immunosuppressive microenvironment (67). In head and neck squamous cell carcinoma (HNSCC), multiplex immunofluorescence analysis revealed that *CMTM4* expression was significantly positively correlated with the density of CD8^+^ T cells and PD-1^+^ immune cells in the TME. These findings suggest that *CMTM4*-high tumors may display an “inflamed but suppressed” immune phenotype, characterized by immune-cell infiltration without effective cytotoxic function. However, this remains a hypothesis that requires spatial and functional validation (68).

In summary, we hypothesize that *CMTM4* expression indicates a tumor has established an “immunologically suppressed but infiltrated” microenvironment: although CD8^+^ T cells are spatially infiltrated, they are functionally inactivated. This mechanism also provides a reasonable explanation for the seemingly contradictory relationship between *CMTM4* and T-cell infiltration across different cancer types, the critical factor is not the presence or absence of immune cells, but rather the integrity of their function. It should be emphasized that this interpretation is currently a hypothetical inference; it has not yet undergone systematic functional validation across multiple cancer types and cannot fully account for the differences in correlations observed across all cancer types. Further research is needed to confirm these findings.

### 4.3 CD4^+^ T cells

As central regulators of the adaptive immune response, CD4^+^ T cells differentiate into various T helper (Th) cell subsets that play a broad and critical role in anti-tumor immunity by synergistically regulating cellular and humoral immunity (69). However, the TME can induce the formation of immunosuppressive CD4^+^ T cell subsets, thereby attenuating the anti-tumor immune response (5, 70).

Although research on the regulatory mechanisms of *CMTM4* in CD4^+^ T cells is still in its early stages, evidence suggests that its function exhibits significant context-dependence. In LC and BC, knocking down *CMTM4* reprograms MDSCs, polarizing them from a pro-tumor M2-like phenotype to an anti-tumor M1-like phenotype, thereby reshaping the T-cell population: this is manifested by a decrease in the proportion of CD4^+^ T cells and Tregs, while the infiltration of cytotoxic CD8^+^ T cells increases, ultimately reversing the immunosuppressive microenvironment and activating the anti-tumor immune response (67). In OC, cancer cells deliver CMTM4 to macrophages via exosomes, inducing their polarization toward the M2 phenotype, which suppresses the activation of CD4^+^ T cells (9).

### 4.4 Tregs

Tregs are central mediators of tumor-induced immunosuppression, and their high infiltration typically portends poor patient prognosis. Recent studies have revealed that Tregs in the TME exhibit unique activation phenotypes and tissue-repair functions that extend far beyond their classical immunosuppressive role (71, 72).

Although studies directly investigating the interaction between *CMTM4* and Tregs are limited, *CMTM4* has been shown to play a role in shaping the immunosuppressive microenvironment, which may indirectly influence the homeostasis and function of Tregs. In LC and BC, downregulation of *CMTM4* expression leads to a decrease in the proportion of Tregs; this mechanism effectively resolves Treg-mediated immunosuppression, reverses the immune balance of the TME, and thereby activates a robust anti-tumor immune response (67). In OC, cancer cells deliver *CMTM4* to macrophages via exosomes, inducing their polarization to the M2 phenotype, secreting immunosuppressive factors such as TGF-β1, and increasing the number of Tregs. The increased number of Tregs further enhances the suppression of CD8^+^ and CD4^+^ T cells, collectively shaping a deeply suppressed TME, completely dismantling the anti-tumor T-cell immune response, and promoting tumor immune evasion (9). In ESCA tissues, *CMTM4* expression levels are positively correlated with Tregs in the TME, indicating that *CMTM4* contributes to the immunosuppressive microenvironment of ESCA by regulating Tregs (11).

### 4.5 Dendritic cells

DCs are key to initiating anti-tumor T-cell immunity. In recent years, research has shifted toward specific DC subsets, particularly cDC1, which is indispensable for cross-presentation of tumor antigens and driving CD8^+^ T-cell responses (73). The TME can lead to DCs dysfunction, thereby preventing the effective initiation of immune responses (74).

Currently, there are few studies on the direct regulation of DCs by *CMTM4*; however, the negative correlation between its expression and DC infiltration suggests a potential regulatory relationship. In HCC, *CMTM4* expression is significantly negatively correlated with the level of DCs infiltration (6, 65). This finding suggests that high *CMTM4* expression may potentially inhibit the recruitment or functional activity of DCs within the TME, which in turn may affect subsequent antigen presentation and T-cell immune activation processes.

### 4.6 Myeloid-derived suppressor cells

MDSCs are another key group of immunosuppressive cells in the TME, and their accumulation is closely associated with tumor progression and resistance to immunotherapy. Recent single-cell analyses have revealed the complex heterogeneity and differentiation pathways of MDSCs in human cancers (75).

**Figure 4:**
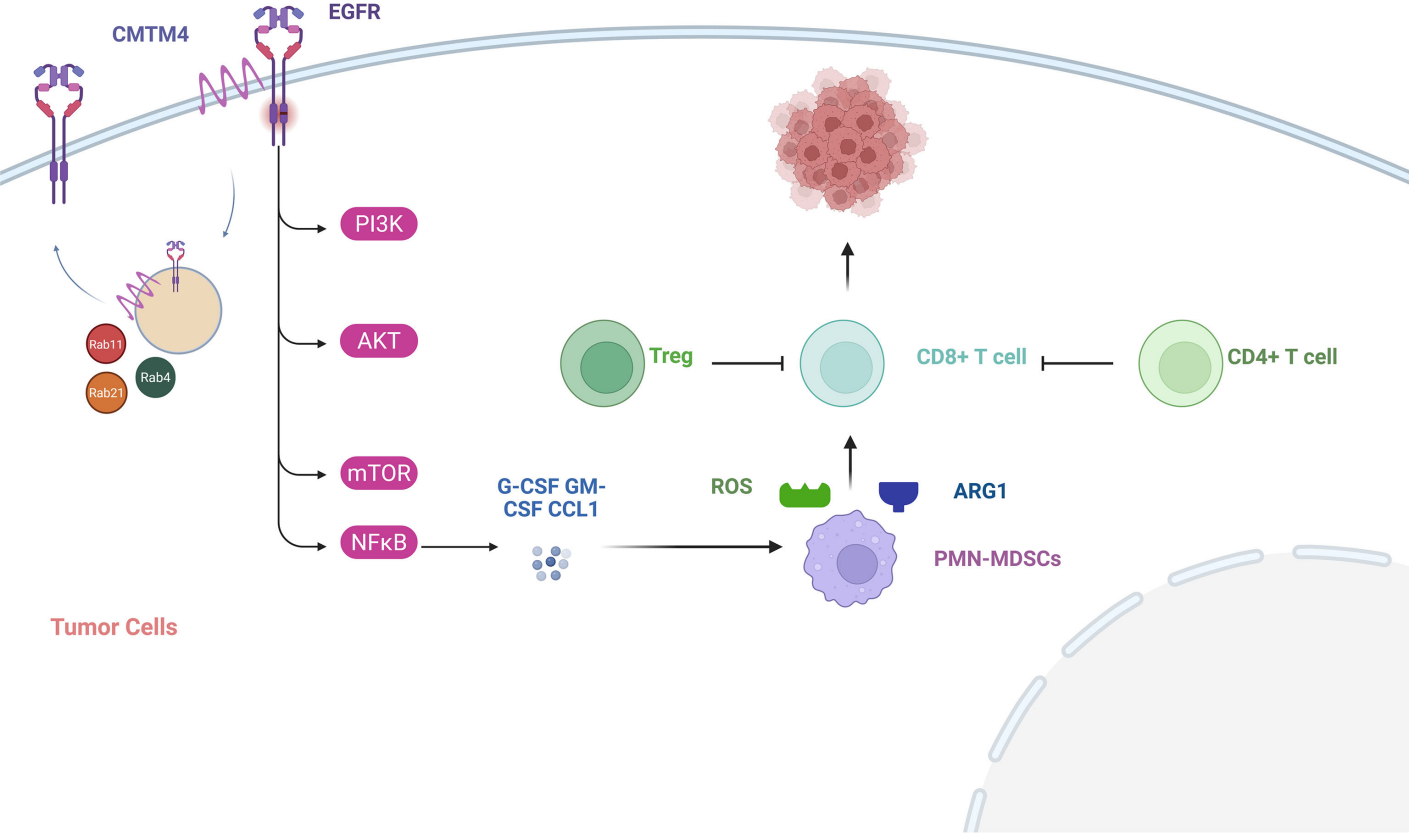
CMTM4-associated EGFR trafficking, MDSC recruitment, and therapeutic intervention points. CMTM4 can regulate EGFR trafficking and downstream inflammatory signaling, leading to recruitment and activation of MDSCs. These MDSCs suppress cytotoxic CD8^+^ T-cell function and promote an immunosuppressive TME. This pathway provides potential intervention points, including CMTM4-related targeting, EGFR inhibitor sensitization, MDSC modulation, and combination with PD-1/PD-L1 blockade. These strategies remain largely preclinical and require further validation.

In lung cancers, *CMTM4* stabilizes EGFR and promotes its Rab4, Rab11, Rab21, and other GTPase-dependent membrane cycling. This continuously activates downstream PI3K/Akt/mTOR and NF-κB signaling pathways, driving tumor cells to secrete inflammatory mediators such as G-CSF, GM-CSF, and CCL1. These mediators recruit and polarize PMN-MDSCs with immunosuppressive functions. These MDSCs significantly suppress CD8^+^ T cell tumor infiltration and cytotoxic function through mechanisms including arginase 1 (ARG1)-mediated metabolic reprogramming and reactive oxygen species (ROS) production. Concurrently, they increase the proportion of CD4^+^ T cells and Tregs, thereby establishing an immunosuppressive microenvironment that promotes tumor progression. Targeting *CMTM4* reverses these processes, enhances EGFR inhibitor sensitivity, and may improve the efficacy of immune checkpoint blockade in experimental models (67). **Figure 4** highlights a checkpoint-independent pathway in which *CMTM4* regulates EGFR trafficking and inflammatory signaling, thereby promoting MDSC recruitment and suppressing CD8^+^ T-cell-mediated anti-tumor immunity. This pathway provides a rationale for combining *CMTM4*-related intervention with immunotherapy or EGFR-targeted therapy in selected preclinical models, although clinical validation is still required. In cervical cancer (CC), CMTM4 acts as a molecular hub for recruiting MDSCs by targeting PHB2 and activating the downstream STING/TBK1/STAT6 signaling axis. This process primarily relies on the CCL2/CCR2 chemotactic axis for cell recruitment and promotes MDSC differentiation and immunosuppressive functions via the IL-6/GP130 signaling pathway, ultimately accelerating tumor progression (76).

### 4.7 Cancer stem cells

CSCs are cells within tumor tissues that possess stem cell characteristics, including multipotent differentiation potential, self-renewal, and tumorigenic capacity. CSCs can regulate the function of stromal cells in the TME, promote tumor progression, and simultaneously suppress immune cell-mediated elimination (77, 78). In HNSCC, studies have shown that downregulation of *CMTM4* significantly attenuates cancer stem cell properties by inhibiting the AKT signaling pathway, as evidenced by decreased expression levels of stem cell-associated markers SOX2, CD44, BMI1, and ALDH1 (68). Previous studies have shown that *CMTM4* can continuously activate downstream AKT/mTOR and NF-κB signaling pathways by regulating the cycling and stability of EGFR, thereby enhancing the malignant phenotype of LC cells and reducing their sensitivity to targeted drugs. Although this study did not directly define CSCs, both AKT/mTOR and NF-κB are core pathways for maintaining tumor stem cell stemness, self-renewal, and drug resistance, suggesting that *CMTM4* may participate in the maintenance of stemness and regulation of drug resistance in LC stem cells through the aforementioned signaling axes (67).

### 4.8 Conceptual framework of *CMTM4*-mediated tumor immune regulation

Taken together, current evidence suggests that *CMTM4* participates in tumor immune regulation through three interconnected layers. First, it contributes to checkpoint-protein trafficking and membrane stability, particularly in *CMTM6*-deficient or *CMTM6*-low contexts. Second, it shapes the immune compartment of the TME by influencing TAM polarization, MDSC recruitment, Treg enrichment, and CD8^+^ T-cell dysfunction. Third, *CMTM4*-related pathways may provide potential intervention points, including *CMTM4/CMTM6* co-targeting, immune checkpoint blockade, myeloid-cell modulation, EGFR-related sensitization, and radiosensitization. However, these strategies remain largely preclinical and require prospective validation.

## 5 Research progress on *CMTM4* and cancer immunotherapy

Cancer immunotherapy has become an important component of cancer treatment, yet only a subset of patients achieve durable clinical benefit. Based on the mechanisms discussed above, *CMTM4* has attracted attention as a molecule that may connect membrane protein trafficking, immune-cell remodeling, and treatment resistance. However, its translational value remains incompletely established. Current evidence is largely derived from cell-based experiments, animal models, retrospective tissue analyses, and bioinformatic cohorts. Therefore, *CMTM4* should currently be considered a candidate biomarker and investigational therapeutic target rather than a clinically validated marker or established target.

### 5.1 Potential *CMTM4*-directed single-target strategies

*CMTM4*-directed single-target strategies may be most relevant in tumor contexts where *CMTM4* contributes to malignant progression or treatment resistance through tumor-cell-intrinsic or inflammatory signaling pathways. Rather than functioning solely as a regulator of immune checkpoint signaling, *CMTM4* may participate in EMT regulation, tumor-cell motility, inflammatory signal transduction, cancer stem cell–related phenotypes, angiogenesis, and resistance to conventional therapies in selected tumor models. These findings suggest that *CMTM4*-targeted intervention may have therapeutic relevance beyond immune checkpoint modulation. In HNSCC, its regulation of EMT exhibits tissue specificity, promoting the EMT process via the SNAIL/MMP2 signaling axis (28, 68, 79). Furthermore, *CMTM4* can participate in tumor progression through mechanisms such as regulating tumor stem cell properties and angiogenesis, further highlighting its multi-target regulatory advantages (67, 80).

The therapeutic value of *CMTM4* single-target intervention varies across tumor types. Furthermore, as a crucial subunit of the IL-17 receptor, *CMTM4* participates in Helicobacter pylori-induced chronic gastric mucosal inflammation and malignant transformation by regulating the IL-17RC/NF-κB/NOX1 signaling pathway. Knocking out *CMTM4* inhibits downstream IL-17 inflammatory signaling and slows the malignant progression of the gastric mucosa, offering new insights for the intervention of gastric precancerous lesions and GC (39).

In LARC, *CMTM4* has been reported as a potential biomarker associated with neoadjuvant chemoradiotherapy (nCRT) response and prognosis. Data from 228 LARC patients show that patients with high CMTM4 expression prior to nCRT had poorer treatment outcomes; expression levels were significantly lower in the pathological complete response (pCR) group compared to the non-pCR group. Furthermore, patients with low *CMTM4* expression had better disease-free survival (DFS) and OS, making *CMTM4* an independent prognostic factor for DFS; *In vitro* experiments further confirmed that knocking down *CMTM4* can reverse radiation resistance in CRC cells, highlighting its potential as a radiosensitizing target for chemoradiotherapy (64).

Overall, *CMTM4*-directed single-target intervention may be more appropriate for tumors in which *CMTM4* plays a dominant role in tumor-cell-intrinsic progression, inflammatory signaling, or treatment resistance. However, current evidence remains largely preclinical or retrospective. Therefore, the therapeutic value of *CMTM4* single-target strategies requires further validation using standardized detection methods, molecularly stratified patient cohorts, and prospective clinical studies.

### 5.2 Potential *CMTM4*-based combination strategies

Current preclinical and translational evidence suggests that CMTM4-based combination strategies may enhance anti-tumor effects in selected tumor models. However, these findings should be interpreted cautiously because most evidence is derived from cell experiments, animal models, or retrospective clinical analyses. No prospective clinical trial has yet established *CMTM4*-directed combination therapy as a standard therapeutic strategy. In experimental models of hepatocellular carcinoma and intrahepatic cholangiocarcinoma, *CMTM4* inhibition enhanced T-cell-mediated anti-tumor immunity and increased sensitivity to PD-L1 blockade. These findings provide a mechanistic rationale for combining *CMTM4*-directed intervention with immune checkpoint blockade. However, this strategy remains preclinical, and whether it can overcome immune checkpoint inhibitor resistance in patients has not been established (7). Two independent immunotherapy cohort analyses showed that patients with high *CMTM4*-related checkpoint scores and high CD4^+^ T-cell infiltration had higher objective response rates to immunotherapy. In the IMvigor210 cohort, the objective response rate was 28.4% in the high-score group and 17.8% in the low-score group; in the GSE176307 cohort, the corresponding rates were 21.4% and 15.5%, respectively. These high-score groups also showed improved survival in both cohorts (IMvigor210, P = 0.032; GSE176307, P = 0.042). These findings suggest that the predictive value of *CMTM4* should be interpreted together with immune infiltration status rather than as a single independent marker (66).

In GC, the co-expression of CMTM6/4 and PD-L1 on the membrane of tumor epithelial cells is significantly positively correlated with the short-term efficacy of anti-PD-1/PD-L1 immunotherapy. Its predictive value outperforms that of single PD-L1 detection, supporting its potential as a candidate biomarker for immunotherapy response. Experimental data show that, in a cohort of 48 patients receiving immunotherapy, those with a partial response (PR) had significantly higher expression levels of CMTM6, CMTM4 and PD-L1 than those in the stable disease (SD) and progressive disease (PD) groups (*p* = 0.043, *p* = 0.0199, and *p =* 0.0341, respectively), and that the proportion of CMTM6/4 double-positive cells was significantly higher in the PR group than in the PD group (*p* = 0.013) (52).

Several preclinical studies have reported that CMTM4-directed intervention combined with immune checkpoint blockade or other immunomodulatory strategies may produce stronger anti-tumor effects than CMTM4-directed intervention alone in selected cancer models, including CC, OC, LC, and BC. However, these findings were obtained from different experimental systems, and the magnitude of benefit should not be directly extrapolated to clinical efficacy (9, 67, 76).

To further clarify the translational relevance of *CMTM4* across tumor types, available clinical and translational studies are summarized in **Table 1**. This table lists cancer type, evidence type, sample size or model, major *CMTM4*-related findings, clinical or translational outcomes, and evidence level.

**Table 1:** Clinical and translational evidence of *CMTM4* in human cancers.

Cancer type	Evidence type	Sample size/model	Main CMTM4-related finding	Clinical/translational outcome	Evidence level
LARC	Retrospective clinical cohort + *in vitro*	228 patients; CRC cell experiments	High pre-nCRT CMTM4 expression was associated with poorer nCRT response	Lower pCR; worse DFS/OS; independent DFS factor, HR 1.759	Retrospective + experimental
GC	Retrospective translational cohort	48 pre-immunotherapy patients; tissue microarray; TCGA/scRNA-seq	Membrane CMTM6/4-PD-L1 co-expression on tumor epithelium was associated with anti-PD-1/L1 response	Better short-term response; OS association	Retrospective/translational
HCC/ICC	Translational + preclinical	TCGA/HKU-QMH datasets; HCC/ICC cell lines; mouse models	CMTM4 stabilized cell-surface PD-L1 via post-translational mechanisms	Enhanced response to PD-L1 blockade in mouse models	Translational/preclinical
HCC	IHC cohort + bioinformatics	90 HCC tissues; TCGA-LIHC; Imvigor210; GSE176307	CMTM4/PD-L1/CD4 composite status was associated with prognosis and immune features	Prognostic association; potential ICI-response relevance	Retrospective/bioinformatic
Glioma	Prognostic analysis	Not reported	CMTM4/CMTM6 were evaluated as potential PD-L1-related regulators	Prognostic association	Retrospective/prognostic
HNSCC	Translational + *in vitro*	Not reported	CMTM4 was associated with EMT, PD-L1 expression, CSC-like phenotypes, and AKT signaling	Prognostic/immune association; *in vitro* functional evidence	Retrospective/translational
OC	Translational/preclinical	OC models; exosomal CMTM4/TAM assays	Exosomal CMTM4 induced immunosuppressive TAMs via NF-κB, cytokines, and ICAM1	Promoted metastasis and attenuated anti-PD-1 sensitivity in models	Preclinical/translational
LC/BC	Preclinical	Lung and mammary tumor models	CMTM4 promoted EGFR recycling and inflammatory signaling linked to MDSC recruitment	Enhanced immune suppression and drug resistance in models	Preclinical
CC	Preclinical/translational	CC models; MDSC assays	CMTM4 stabilized PHB2 and activated STING/TBK1/STAT6, inducing CCL2/IL-6-mediated MDSC recruitment	CMTM4 inhibition enhanced anti-PD-1 response in models	Preclinical/translational

As shown in **Table 1**, current clinical evidence for *CMTM4* remains largely retrospective, correlative, or exploratory. Although *CMTM4* shows potential as a candidate biomarker, its clinical utility requires validation in prospective and independently designed cohorts.

Based on the available mechanistic and translational evidence, the key characteristics of *CMTM4*-directed single-target strategies and *CMTM4*-based co-targeting approaches are summarized in **Table 2**. This comparison highlights the theoretical basis, applicable contexts, potential advantages, limitations, and current evidence level of each strategy.

**Table 2:** Comparative analysis of *CMTM4* single-target and co-target strategies.

Feature	Single-target therapy(CMTM4 single-target)	Co-target therapy (CMTM4+CMTM6 or CMTM4+PD-1/PD-L1)
Theoretical Basis	CMTM4 acts as a major stabilizer of PD-L1 in certain tumors(e.g., low CMTM6 expression/loss of function) and regulates non-PD-L1-dependent immune suppression pathways (such as MDSC recruitment and IL-17 signaling).	CMTM4 and CMTM6 form a functionally redundant PD-L1 stabilization network; targeting a single molecule may lead to resistance due to compensatory upregulation of the other molecule. Combined PD-1/PD-L1 blockade could synergistically relieve T cell suppression.
Applicable Scenarios	Tumors with low CMTM6 expression or loss of function (e.g., some GC, HCC subtypes); Tumors with active CMTM4-mediated non-PD-L1-dependent immunosuppressive pathways (e.g., IL-17-driven precancerous lesions/gastric cancer);Patients requiring sensitization with conventional therapy (e.g., radiosensitization in LARC).	Solid tumors co-expressing CMTM4 and CMTM6 (e.g., HCC, GC, LC, BC, OC, CC, etc.) “Cold tumors” with inherent resistance to PD-1/PD-L1 inhibitors and high CMTM4/6 expression. Patients with immune tolerance and ICI resistance to be overcome.
Key Advantage	Simpler regimen; potential as a sensitizer for conventional therapies (e.g., radiotherapy in LARC).	Overcomes immune tolerance and ICI resistance; achieves synergistic anti-tumor effects. The tumor inhibition rate is increased to approximately 65%–72% (compared to approximately 30%–40% for single-target in preclinical studies)
Limitation	The efficacy is limited (partial inhibition, difficult to complete remission); There may be a compensatory upregulation of CMTM6 leading to drug resistance. The effect on low PD-L1-dependent tumors is uncertain.	Increased complexity; potential for overlapping toxicities; requires careful patient selection.
Level of preclinical evidence	Preclinical studies across multiple tumor models have reported approximately 30%–40% tumor growth inhibition and approximately 15%–20% survival extension with Cmtm4 single-gene knockout, but complete remissions are difficult to achieve.	In CC, LC, BC, OC and other models, CMTM4-targeted therapy combined with PD-1/PD-L1 inhibitors increased tumor response rates to approximately 65%–72% (e.g., a 72% reduction in OC metastases), and some models achieved complete remission.

Overall, *CMTM4*-directed intervention and *CMTM4*-based combination strategies should be regarded as investigational approaches with potential translational relevance in selected tumor contexts. Current evidence supports further investigation of *CMTM4* in relation to checkpoint dependency, myeloid-cell modulation, macrophage polarization, and treatment resistance. However, standardized detection methods, patient stratification criteria, and prospective clinical validation are required before *CMTM4* can be used as a clinical biomarker or therapeutic target.

## 6 Unresolved questions, future hypotheses, and translational challenges

Although accumulating evidence supports the involvement of *CMTM4* in tumor immune regulation and tumor cell biology, several fundamental mechanistic and translational challenges remain unresolved. These challenges extend beyond immune checkpoint regulation and involve the broader functions of *CMTM4* in the TME.

First, the biological function of *CMTM4* is highly context-dependent. As discussed above, although *CMTM4* can contribute to PD-L1 protein stability in tumor epithelial cells, the negative correlation between *CMTM4* and PD-L1 in the stromal compartment of GC indicates that this mechanism cannot be generalized across all tumor compartments. This contradiction highlights the limitations of interpreting tissue-level correlations as direct functional regulation. Future studies should further distinguish tumor-cell-, stromal-cell-, and immune-cell-specific *CMTM4* functions using spatial and single-cell approaches.

Second, several mechanistic aspects of *CMTM4* biology remain insufficiently defined. The upstream regulatory network controlling *CMTM4* expression is still unclear. Existing evidence suggests that non-coding RNAs and epigenetic alterations, including promoter methylation, may influence *CMTM4* expression. However, whether inflammatory cytokines, hypoxia, metabolic stress, oncogenic signaling, or immune-cell-derived factors directly regulate *CMTM4* requires further investigation. Clarifying these upstream mechanisms may help explain the tumor-type-specific and compartment-dependent functions of *CMTM4*.

The structural basis of *CMTM4*-mediated checkpoint regulation also remains unresolved. Although *CMTM4* contains a conserved MARVEL transmembrane domain and participates in membrane-associated protein trafficking, high-resolution evidence defining its precise binding interface with PD-L1 is still lacking. Therefore, current evidence supports a functional relationship between *CMTM4* and checkpoint-protein stability but does not allow definitive conclusions regarding specific binding domains. Future studies using mutagenesis mapping, proximity labeling, cross-linking mass spectrometry, and structural biology approaches may help determine whether *CMTM4* directly associates with PD-L1 through transmembrane regions or requires additional adaptor proteins.

Furthermore, the post-translational regulation of *CMTM4* itself remains poorly understood. Current studies have focused mainly on how *CMTM4* affects checkpoint-protein stability, whereas the regulation of *CMTM4* after translation is less clear. Whether *CMTM4* undergoes functionally relevant phosphorylation, ubiquitination, glycosylation, palmitoylation, or lysosomal turnover remains unknown. Addressing these questions will be important for understanding *CMTM4* stability, localization, interaction partners, and therapeutic druggability.

Third, existing research models have significant limitations in reproducing the true biological functions of *CMTM4*. Most of the mechanistic studies in this paper rely on immortalized cell lines and genetically engineered mouse models, which struggle to simulate the high heterogeneity of the human TME and the dynamic interactions of immune cells. Consequently, the specific functions of *CMTM4* in different immune cell subsets (e.g., M1 vs. M2 macrophages, exhausted vs. functional CD8^+^ T cells) and its role in different tumor subtypes have not been sufficiently studied, limiting our ability to tailor single-target and co-target therapies to specific patient populations.

Finally, from the perspective of clinical translation, the therapeutic efficacy of *CMTM4*-targeted monotherapy and combination therapy exhibits cancer type-specific heterogeneity. For instance, CMTM4-directed intervention has shown potential as a radiosensitizing strategy in LARC-related experimental settings, whereas combination strategies with immune checkpoint blockade have demonstrated enhanced anti-tumor effects mainly in preclinical models of LC, BC, OC, and CC. These significant contextual differences require us to make comprehensive assessments of *CMTM4’s* clinical significance by considering specific tissue types, molecular subtypes, and spatial distribution(e.g., epithelial vs. stromal expression in GC), rather than simply generalizing it as a universal biomarker or target. Notably, most existing evidence for *CMTM4* is derived from preclinical models and single-center retrospective clinical studies, and large-scale prospective clinical validation is still lacking to confirm the generalizability of these findings for routine clinical application. To date, no CMTM4-directed interventional clinical trial has established CMTM4 as a standard biomarker or therapeutic target in oncology. Meanwhile, current clinical research still lacks precise evaluation criteria tailored to different cancer types, which remains a core obstacle to its clinical translation.

## 7 Summary and expectations

Immunotherapy achieves tumor killing by modulating the TME to activate and enhance the body’s innate immune effector cells, a mechanism that has revolutionized traditional cancer treatment. As a molecule linking innate and adaptive immunity, *CMTM4* plays a complex and vital role in this process. Its functions extend beyond immune checkpoint regulation and include tumor proliferation, EMT, angiogenesis, cancer stem cell–related phenotypes, and sensitization to radiotherapy or chemotherapy. The functional relationship between *CMTM4* and its homolog *CMTM6* further expands the tumor immune regulatory network and provides new directions for target selection in immunotherapy. Meanwhile, the potential value of *CMTM4* as a candidate biomarker and potential therapeutic target has been suggested in multiple cancer types; however, most available evidence remains retrospective, correlative, or preclinical. As discussed above, research on *CMTM4* remains hampered by conflicting mechanisms, model limitations, and uncertainties in translation.

Based on the existing research foundation and current challenges, future studies should focus on the following areas:

1. Utilize proteomics, gene-editing approaches, proximity labeling, mutagenesis mapping, and structural biology methods to identify upstream regulators, interaction partners, and potential post-translational regulatory mechanisms of *CMTM4* in different cellular contexts. These approaches may help clarify the structural basis of *CMTM4*-mediated checkpoint regulation and explain the functional heterogeneity of *CMTM4* across tumor types and cellular compartments.
2. Promote research systems that more closely mimic human physiology, such as patient-derived organoids and humanized mouse models, and combine these with single-cell multi-omics technologies to resolve the function of *CMTM4* in specific immune cell subsets at high resolution.
3. Expand the investigation of *CMTM4’s* regulation of other key membrane molecules, particularly immune-activating and immune-suppressive receptors. Future studies should evaluate the druggability of *CMTM4* as a membrane-associated protein, including extracellular accessibility, structural targeting feasibility, tumor-selective delivery, potential toxicity, and compensatory effects of *CMTM6*. RNA-based approaches, antibody-based strategies, or combination regimens may be explored only after these issues are clarified.
4. Establish precise evaluation criteria for *CMTM4* across different cancer types, clarify its applicability as a biomarker, and determine the optimal timing and combination regimens for intervention as a therapeutic target, thereby facilitating its translation from basic research to clinical application.

In summary, as a molecule linking tumor cells to the tumor immune microenvironment, *CMTM4* may hold translational potential as a candidate biomarker and therapeutic target, but its clinical application requires rigorous prospective validation. Addressing the aforementioned challenges in mechanistic research, model systems, and clinical translation will be critical to translating the promise of *CMTM4*-targeted strategies into clinical practice, ultimately advancing personalized cancer immunotherapy and improving patient outcomes.
